# Cumulative Risk Meets Inter-Individual Variability: Probabilistic Concentration Addition of Complex Mixture Exposures in a Population-Based Human In Vitro Model

**DOI:** 10.3390/toxics10100549

**Published:** 2022-09-20

**Authors:** Suji Jang, Lucie C. Ford, Ivan Rusyn, Weihsueh A. Chiu

**Affiliations:** 1 Interdisciplinary Faculty of Toxicology, College of Veterinary Medicine and Biomedical Sciences, Texas A&M University, College Station, TX 77843, USA; 2 Department of Veterinary Physiology and Pharmacology, College of Veterinary Medicine and Biomedical Sciences, Texas A&M University, College Station, TX 77843, USA

**Keywords:** cumulative risk, dose addition, concentration addition, inter-individual variability, toxicodynamics, chemical mixtures, defined mixtures, human health risk assessment, uncertainty factors, new approach methods

## Abstract

Although humans are continuously exposed to complex chemical mixtures in the environment, it has been extremely challenging to investigate the resulting cumulative risks and impacts. Recent studies proposed the use of “new approach methods,” in particular in vitro assays, for hazard and dose–response evaluation of mixtures. We previously found, using five human cell-based assays, that concentration addition (CA), the usual default approach to calculate cumulative risk, is mostly accurate to within an order of magnitude. Here, we extend these findings to further investigate how cell-based data can be used to quantify inter-individual variability in CA. Utilizing data from testing 42 Superfund priority chemicals separately and in 8 defined mixtures in a human cell-based population-wide in vitro model, we applied CA to predict effective concentrations for cytotoxicity for each individual, for “typical” (median) and “sensitive” (first percentile) members of the population, and for the median-to-sensitive individual ratio (defined as the toxicodynamic variability factor, TDVF). We quantified the accuracy of CA with the Loewe Additivity Index (LAI). We found that LAI varies more between different mixtures than between different individuals, and that predictions of the population median are generally more accurate than predictions for the “sensitive” individual or the TDVF. Moreover, LAI values were generally <1, indicating that the mixtures were more potent than predicted by CA. Together with our previous studies, we posit that new approach methods data from human cell-based in vitro assays, including multiple phenotypes in diverse cell types and studies in a population-wide model, can fill critical data gaps in cumulative risk assessment, but more sophisticated models of in vitro mixture additivity and bioavailability may be needed. In the meantime, because simple CA models may underestimate potency by an order of magnitude or more, either whole-mixture testing in vitro or, alternatively, more stringent benchmarks of cumulative risk indices (e.g., lower hazard index) may be needed to ensure public health protection.

## 1. Introduction

Humans are continuously exposed to complex chemical mixtures in the environment; however, progress in establishing standardized experimental and/or modeling approaches to quantifying the resulting cumulative risks and impacts has been limited. With few exceptions, such as diesel exhaust or particulate matter [1,2], there has been a dearth of toxicity data on whole mixtures, in large part due to their infinite diversity. Additionally, for both drugs and environmental chemicals, either mechanistic or empirical understanding of chemical interactions is limited to situations where there are only a handful of components (e.g., drug–drug interactions or chemical interaction profiles) [3,4]. As a result of these challenges, component-based methods have been the mainstay of mixtures and cumulative risk assessment, with dose or concentration addition (DA or CA) being the “default”, presumably health-protective, approach [5,6,7]. DA/CA assumes that any component of a mixture can be replaced by a proportional amount of another chemical without changing the overall response, with the proportion determined by their relative potencies [8]. This concept was originally derived from pharmacological principles relating to ligand binding theory [9,10,11], but has since come into widespread use. For instance, DA/CA is the basis for most chemical or mechanistic class-based approaches to cumulative risk (e.g., polycyclic aromatic hydrocarbons [PAHs], aryl hydrocarbon receptor [AhR] agonists, carbamates) [12,13,14]. Additionally, many component-based chemical risk assessment approaches for combined exposures are also based on DA/CA, including the Hazard Index (HI = sum of each chemical component’s Hazard Quotient (HQ = Exposure ÷ “Safe Dose”)) and its ecotoxicity equivalent, the Risk Quotient [5,6,11]. 

There has been considerable recent interest and work on CA and other mixture modeling approaches, particularly in the context of in vitro assays and whole-mixture testing. For instance, several studies have found that effects of bioavailable extracts from contaminated sediments are consistent with CA or a model combining CA and independent action [15,16,17]. Additionally, both non-targeted and effect-directed approaches have been proposed to identify components within mixtures [18,19]. Moreover, efforts to incorporate temporal dynamics through the use of toxicokinetic–toxicodynamic (TK–TD) models have been applied in the context of ecotoxicology [20,21,22,23]. However, although addressing human inter-individual variability is a key concern for both single-chemical and mixture risk assessment, there have been only a few efforts, largely limited to pharmacokinetics, to integrate population variability in drug or chemical metabolism into analyses of mixture interactions [24,25,26]. For instance, it has been found that the degree of population variability in drug–drug interactions may vary from negligible (<10%) to twofold [24]. Thus, the accuracy of additivity assumptions and its inter-individual variation remain as key uncertainties in cumulative risk assessment.

There has been increasing interest in using so-called New Approach Methodologies (NAMs), in particular, human cell-based in vitro models, to provide empirical data on mixtures for use in cumulative risk assessment [27]. We have previously demonstrated the feasibility of high-throughput in vitro testing of environmental mixtures [28,29], defined mixtures [30], and complex substances [31,32,33]. In particular, using a diverse set of 42 chemicals from the Agency for Toxic Substances and Disease Registry (ATSDR) substance priority list, we found that in 8 different defined mixtures, CA predictions were typically within an order of magnitude of the effects of defined mixtures, but that the accuracy of additivity assumptions varied greatly by phenotype [30]. Similarly, the emergence of population-based NAMs has opened the door to replacing default assumptions about inter-individual variability with data-derived factors [34,35,36]. For pharmacokinetics, high-throughput toxicokinetic (TK) modeling using in vitro data has been extended to include characterization of population variability across life stages. Additionally, we have shown that populations of either human induced pluripotent stem cell (iPSC)-derived cardiomyocytes [37,38,39,40] or human lymphoblastoid cells (LCLs) [41,42,43,44] may be useful in characterizing inter-individual variability in toxicodynamics (TD). In both of these cases, the goal is to replace the default uncertainty factors for TK or TD variability with data-derived TK or TD variability factors (TKVF and TDVF, respectively) [45,46].

These studies suggest that a population-based in vitro NAM may be useful in investigating the intersection of cumulative risk and population variability of mixtures. Therefore, utilizing data from a panel of 146 LCLs from genetically diverse individuals [47], we apply Bayesian methods to investigate the accuracy of applying CA at either the individual or population level. Specifically, using the same set of 42 ATSDR priority chemicals in 8 defined mixtures as previously investigated [30], we evaluated the accuracy of CA for predicting points of departure (PODs) for each individual separately and for the “typical” (median) and “sensitive” (first percentile) members of the population. Moreover, the ratio between the “typical” and “sensitive” individual can be used as a TDVF to replace the default uncertainty factor. We quantified the accuracy of CA using the Loewe Additivity Index (LAI), and also compared our results to those previously reported in other cell types. Finally, we discuss the implications of these findings for incorporating NAMs into cumulative population risk assessment.

## 2. Materials and Methods

### 2.1. Experimental Designs 

**Figure 1:**
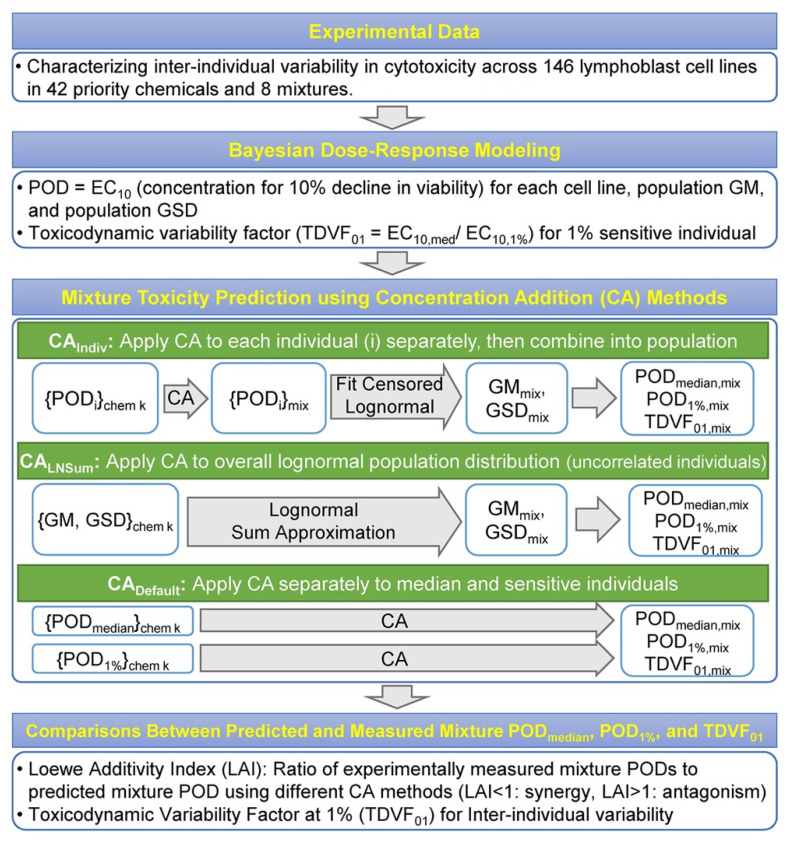
Overall workflow to evaluate population variability in mixture additivity. First, lymphoblastoid cell lines from 146 individuals were exposed in vitro to 42 chemicals and 8 defined mixtures in concentration–response [47]. Cytotoxicity was measured as the endpoint of interest, from which point of departure (POD) and TDVF values were derived using Bayesian concentration–response modeling. Next, the results from individual chemicals were used to predict those of the defined mixtures assuming three different methods for applying concentration addition (CA) to a population, and then compared with the measured POD from the defined mixture experiments. Accuracy was characterized by calculating the Loewe Additivity Index (LAI), which refers to the ratio of the measured and CA-predicted values. Additional details are described in Section 2.

Figure 1 shows the overall workflow of the experiment, data analysis, Bayesian modeling, and CA methods. The detailed methods for the in vitro experiments, including LCL identification, chemical and mixture information, and cytotoxicity data, were previously described [47]. In brief, 146 LCLs were obtained from The Coriell Institute for Medical Research (Camden, NJ) and comprised cells from four subpopulations, three of European descent (Utah residents with European ancestry (CEU), Tuscans in Italy (TSI), British from England and Scotland (GBR)) and one of African descent (Yoruban from Ibadan, Nigeria). Cells were grown in culture and exposed to 42 Superfund priority chemicals and 8 defined mixtures in concentration–response. After a 24 h incubation with the individual chemicals or mixtures, cell viability was assessed using intracellular ATP concentration measured via CellTiter-Glo.

The chemicals were selected to represent potential “real-world” exposures and mixtures, and included a diverse set of environmental pollutants from various chemical classes that included pesticides, high-production-volume chemicals, heavy metals, PAH, and phthalates. As previously detailed [48], the selection was based on the following criteria: (i) chemicals listed on the Agency for Toxic Substances and Disease Registry (ATSDR) priority list; (ii) chemicals commonly detected at Superfund sites; (iii) chemicals representing diverse classes; (iv) chemicals with available human “safe exposure” levels, reverse toxicokinetic and exposure data on U.S. Environmental Protection Agency (U.S. EPA) dashboard, and in vitro data on ToxCast™/Tox21 for comparison [49]. Chemical components along with key chemical properties are provided in Supplementary Table S1, and represent a diversity of chemical classes, hydrophobicity, and ionic strength. After each chemical was first dissolved in 100% cell culture-grade DMSO at a concentration of 20 mM, five 10× serial dilutions were prepared for concentration–response data. Additionally, those samples were further diluted 200× using cell culture media to set the final concentrations as 100, 10, 1, 0.1, or 0.01 μM.

The relative concentrations of mixture components were defined to account for various exposure- and hazard-based scenarios, as described previously in [30], based on the following data: (i) active concentration 50% (AC_50_) from in vitro study data in ToxCast/Tox21 [49]; (ii) points of departure (POD) from in vivo study data used for regulatory reference doses (RfD); (iii) estimates of human exposure levels from ExpoCast [50]; (iv) RfD from in vivo study data [51]. Under each assumption, two mixtures were defined to represent the median (termed “low”) and the upper 95th percentile (termed “high”) for consideration of human toxicokinetic variability. The concentration of each chemical in the group of eight mixtures is provided in Supplementary Table S1. 

### 2.2. Bayesian Modeling of Concentration–Response for Cytotoxicity

Hierarchical Bayesian random-effects Hill models were used to fit a concentration–response model to the cytotoxicity for each chemical and mixture as noted in [44]. It was assumed that the concentration–response data for each chemical or mixture and individual cell line followed a “downward” Hill model [39,44], with an equation with response *y* as a function of concentration *x*, mathematically formulated as follows:

(1) $$y=y_{0}\left(1-\frac{{\left(\frac{x}{x_{0}}\right)}^{n}}{1+{\left(\frac{x}{x_{0}}\right)}^{n}}\right)+\epsilon ,$$

where variable $y_{0}$ is the baseline value, $x_{0}$ is the concentration at a 50% decrease from the baseline response (EC_50_), n is the Hill coefficient, and ϵ is the residual error. Fitting of $y_{0}$ was needed to address “drifting” baseline values even after normalization via vehicle controls. The Hill coefficient was restricted to be ≥1 to prevent atypical fits with very shallow concentration–response curves, as noted previously [30]. The error ϵ between actual and estimated values was assumed to follow a scaled Student’s *t* distribution with scale parameter σ, where ϵ/σ has a standard Student’s *t* distribution, with ν=5 degrees of freedom, to improve robustness for outliers [44].

Based on previous studies [39,40,42,52] and consistent with US EPA guidance for dose–response modeling and determination of the points of departure (PODs), the effective concentration at which a 10% change in cell viability in comparison to control (EC_10_) was selected as the benchmark response [52]. The EC_10_ is related to *x*_0_ and *n* by the equation EC_10_ = *x*_0_ (0.1/0.9)^1/n^, so for computation, we directly sampled from EC_10_ and *n*, and then derived the *x*_0_ for use in Equation 1 using the inverse equation

(2) $$x_{0}=EC_{10}{\left(0.9/0.1\right)}^{1/n}.$$

Additionally, we restricted n≥1 for the Hill coefficient by defining $n=1+\tilde{n}\;$ and restricting $\tilde{n}$ to be non-negative. Specifically, the parameters EC_10_ and $\tilde{n}$ were assumed to have lognormal population variability distributions across individuals (i.e., ln(EC_10_) and $\ln\left(\tilde{n}\right)$ are normal random effects), with uncertain parameters for the population geometric mean (GM) and geometric standard deviation (GSD). Prior distributions for population ln(GM) were normal, and prior distributions for ln(GSD) and for σ were half-normal. 

The Markov chain Monte Carlo (MCMC) algorithm was used to sample the posterior distribution using the R Stan package [53]. For each chemical or mixture, four chains were set and each chain initially contained 4000 iterations. The first half of iterations in each chain were used as warm-up data and were discarded, and the remaining iterations were used for inference. The potential scale reduction factor $\hat{R}$ was used to assess convergence that indicates the adequacy of sampling and to compare between- and within-chain variability. Values of $\hat{R}$ ≤ 1.2 were considered adequately converged [54]. Chain length was doubled until convergence, capping the maximal number of iterations at 16,000 iterations.

### 2.3. Derivation of Points of Departure (PODs) and Toxicodynamic Variability Factor (TDVF_01_)

The EC_10_ was estimated by MCMC sampling for each individual $EC_{10,i}$ and for the population overall (GM_EC10_ and GSD_EC10_). A chemical was considered “inactive” and removed from the database for further data analyses if GM_EC10_ > 3× the maximum tested concentration (e.g., 300 μM for individual chemicals). For evaluating the performance of CA, three predictions were used: the population median EC_10,median_ (=GM_EC10_), the “sensitive” 1st percentile EC_10,1%_ (=GM_EC10_ × GSD_EC10_^−2.326^), and their ratio—the toxicodynamic variability factor at 1% (TDVF_01_ = GSD_EC10_^2.326^ = EC_10,median_/EC_10,1%_) These calculations was repeated for each MCMC sample, so their distributions represent the uncertainty in EC_10,median_, EC_10,1%_, or TDVF_01_ for each chemical or mixture. The generally accepted default uncertainty factor for toxicodynamic variability is 10^1/2^, TDVF = 3.16 [45,46]; this was used as a comparison benchmark. 

### 2.4. Mixture Concentration–Response Prediction Using Concentration Addition Approaches

For the prediction of mixture effects from those of individual-component chemicals, CA and independent action (IA), also known as response additivity, are commonly used. CA is based on an assumption of constant potency ratios across chemicals; therefore, the effective concentration for any effect size *X* (*EC_X_*) for a mixture is the weighted harmonic sum of the *EC_X_* for the components (see review by [55]).

(3) $$\frac{1}{EC_{X,CA}}=\sum \frac{f_{k}}{EC_{X,k}},$$

where $f_{k}$ is the fraction of the *k*th compound in the mixture. While CA was originally based on a premise that all of the components in a mixture act on the same biological site and have the same modes of action, it is now recognized that only a common effect is needed to justify CA [8]. As in previous studies, here we are measuring effects of components and mixtures in the same cell type for the same endpoint, so CA is the appropriate default assumption. Moreover, we previously demonstrated in other cell types that CA showed a greater prediction accuracy with measured PODs compared to those from IA [30]. Furthermore, it has been shown that at low effect sizes, CA and IA tend to coincide [56]. Therefore, here we only assess the accuracy of CA. 

There has been little work on the applicability of CA in the presence of inter-individual variability. We therefore used three different approaches to implement CA in this context, as described in Figure 1, that make different assumptions as to the degree to which sensitive members of the population may respond differently to different components.

Under the first approach, CA_Indiv_, we apply CA to each cell line individually, basically treating them as separate experiments. Thus, CA_Indiv_ uses the individual-level data on inter-individual variability of sensitivity to each component to build the inter-individual distribution of sensitivities for the mixture. The mathematical formula of CA (Equation (3)) is simply applied to each individual *i* and mixture *m*:

(4) $$\frac{1}{EC_{10,CA,m,i}}=\sum_{k=1}^{k_{max}}\frac{f_{k,m}}{EC_{10,k,i}},$$

where $EC_{10,CA,m,i}$ is the CA-predicted EC_10_ of mixture *m* for individual *i*, $f_{k,m}$ is the fraction of the *k*th compound in the mixture (from [30] and Supplementary Table S1), and $EC_{10,k,i}$ is the EC_10_ of chemical *k* for individual *i*. For each mixture, we then fit $EC_{10,CA,m,i}$ across individuals to a censored lognormal distribution to estimate the population GM_EC10CA,m_ and GSD_EC10CA,m_ (censored at 3× the maximum tested concentration) using the fitdistrplus R package. These are then used to calculate the CA-predicted EC_10CA,m,median_, EC_10CA,m,1%_, and TDVF_01CA,m_ for each mixture. To quantify the accuracy of CA, we use the Loewe Additivity Index (LAI), which is the ratio between the measured value and the CA-predicted value [57,58,59,60]. For instance, for the EC_10_ for mixture *m*, individual *i* is:

(5) $$LAI_{m,i}=EC_{10,m,i}/EC_{10,CA,m,i},$$

which is the ratio between the measured and CA-derived values. LAI < 1 indicates greater-than-additive effects (“synergy”), so CA is not protective, while LAI > 1 indicates less-than-additive effects (“antagonism”), so CA is health-protective. The LAI was also calculated for the population median, sensitive 1st percentile, and the TDVF_01_. It should be noted as well that the LAI value is independent of differences in bioavailability across chemicals, as long as the bioavailability is the same across concentrations and mixtures (see Appendix A). 

A second approach, denoted CA_LNsum_, only uses the lognormal summary statistics for each component *k* (i.e., GM_k_ and GSD_k_ of the EC_10_), therefore assuming that individual sensitivities are randomly distributed in an uncorrelated manner across components. Specifically, in this case, the individuals in Equation (4) are independent and identically distributed lognormally, so the GM and GSD are sufficient statistics. Because the lognormal sum does not have a simple closed form solution, we make the common approximation that this sum is also approximately lognormally distributed using the *lognorm R* package [61,62]. Specifically, for each mixture *m*, we define a log-scale mean (µ) and standard deviation (σ) of each component *k* as $\mu_{k,m}=\ln\left(f_{k,m}/GM_{k}\right)$ $\sigma_{k,m}=\ln\left(GSD_{k}\right)$. Using the lognormal sum approximation, we obtain the log-scale mean µ_CA,m_ and standard deviation σ_CA,m_ for the summation. These are then converted to the $GM_{CA,m}=1/\exp\left(\mu_{CA,m}\right)$ and $GSD_{CA,m}=\exp\left(\sigma_{CA,m}\right)$ of the EC_10_ values, from which the population median, sensitive 1st percentile, and TDVF_01_ based on CA are derived and compared to the measured values using the LAI. 

The third approach, denoted CA_default_, is modeled after default dose addition approaches in risk assessment, where the components’ RfDs are used as an inverse weighting factors in calculating a cumulative HI $=\sum Exposure_{k}/RfD_{k}$. In particular, CA_Default_ assumes that sensitivity is the same across each component, so that the most sensitive individual for one component is also the most sensitive for other components. Thus, the mixture median EC_10_ and 1st percentile EC_10_ are derived by separately applying CA to the median and 1st percentile EC_10_ values:

(6) $$\frac{1}{EC_{10,CA,median}}=\sum_{k=1}^{k_{max}}\frac{f_{k,m}}{EC_{10,median,k}},$$

(7) $$\frac{1}{EC_{10,CA,1st}}=\sum_{k=1}^{k_{max}}\frac{f_{k,m}}{EC_{10,1st,k}}$$

The CA-based TDVF_01_ is then derived by taking the ratio.

### 2.5. Data Processing and Reproducibility

All data analysis, modeling, and visualizations are performed using *R* (version 4.1.2) and *RStudio* (version 2022.02.3). The *rstan* package (version 2.26.9) was used for MCMC simulations for dose–response fitting under the operating system of Windows 11. All raw data and codes for checking reproducibility are available in GitHub (https://github.com/Suji-Jang/LCL-2022, accessed on 29 August 2022). More details are provided in both the Supplementary Materials and the GitHub repository.

## 3. Results

### 3.1. Population Variability in Accuracy of Concentration Addition across Individuals

**Figure 2:**
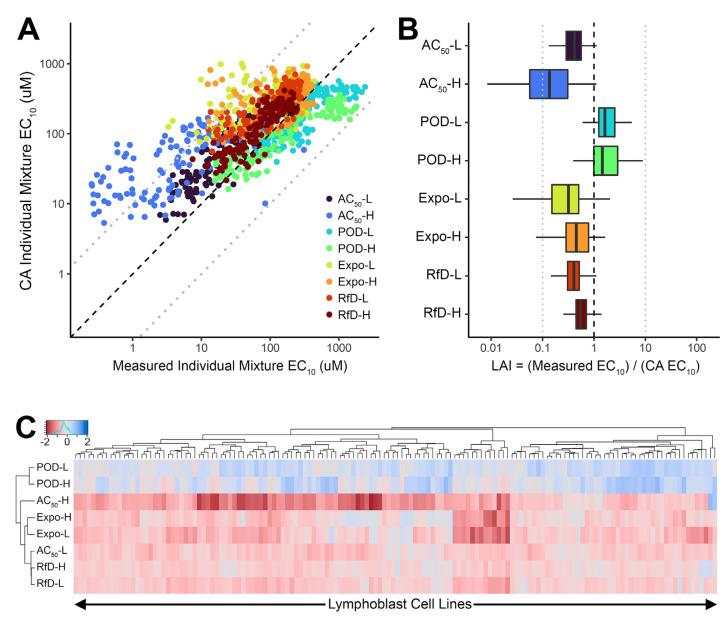
Comparison of mixture points of departure (POD = EC_10_) measured and predicted by concentration addition (CA) for each individual cell line. (**A**)**.** Scatter plot of the median Bayesian posterior estimate of measured and CA-predicted EC_10_. (**B**). Distribution across individuals of Loewe Additivity Index (LAI) for each mixture. (**C**). Heatmap and hierarchical clustering of log_10_(LAI) across mixtures and individual cell lines. In panels (**A**,**B**), dashed line indicates equality, and light dotted lines indicates 10-fold differences in either direction. In panels (**B**,**C**), LAI < 1 (or log_10_(LAI) < 0) indicates greater-than-additive effects (“synergy”) and LAI > 1 (or log_10_(LAI) > 0) indicates less-than-additive effects (“antagonism”).

Figure 2 shows the results of applying CA at the individual level, where each cell line is treated as independent for the purposes of mixture effects. The scatterplot comparing CA-predicted and measured individual EC_10_ (Figure 2A) shows that they have a fair correlation (r^2^ = 0.55), with 92.7% of the points located within a 10-fold boundary (dashed lines). Most of the CA EC_10_s (75.3%) are greater than the measured EC_10_, indicating that CA is likely to be less protective; however, the POD-L and POD-H mixtures tend to have the opposite. The AC_50_-H mixture has the smallest measured EC_10_ as well as the largest inter-individual variation. These results are corroborated in Figure 2B, where LAI < 1 for all mixtures except POD-L and POD-H. The lower and upper quartiles, excluding the AC50-H mixture, are within 10-fold of LAI = 1, with LAI values across all the cell lines and mixtures ranging from 0.25 to 2400.

We also investigated whether there were any clustering patterns among individuals as shown in the heatmap in Figure 2C. The clustering of mixtures shows the same pattern as Figure 2A,B. Interestingly, a few cell lines, such as CEU-NA12827 and GBR-HG00132, had relatively small LAI values, and other specific cell lines such as TSI-NA20809 and TSI-NA20544 had relatively large LAI values across the mixtures. Analysis of variance (ANOVA) showed that mixtures and individuals together explain a significant amount of variation in log_10_ LAI values (*R*^2^ = 0.69, or 69% of the variance explained), though most of the variability in LAI is due to mixtures ($\eta^{2}=0.56$, or 56% contribution) rather than individuals ($\eta^{2}=0.13$, or 13% contribution), with a residual standard error of 10^0.29^.

### 3.2. Comparison of Concentration Addition Approaches for the Median and the Sensitive (First Percentile) Individuals

**Figure 3:**
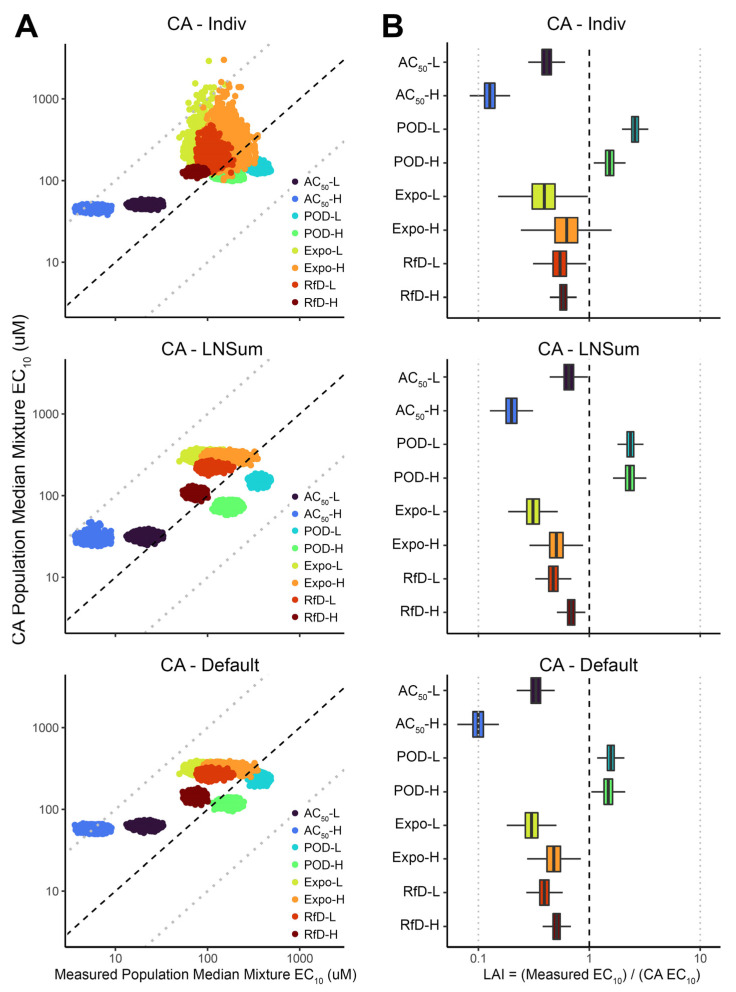
Comparison of mixtures’ points of departure (POD = EC_10_) measured and predicted by different concentration addition (CA) approaches for the population median individual. (**A**) Scatter plot of Bayesian posterior samples of measured and CA-predicted EC_10_ for the population median individual. (**B**) Posterior distribution of Loewe Additivity Index (LAI) for each mixture for the population median individual, where LAI < 1 indicates greater-than-additive effects (“synergy”) and LAI > 1 indicates less-than-additive effects (“antagonism”). In both panels, dashed line indicates equality, and light dotted lines indicates 10-fold differences in either direction. See Materials and Methods and Figure 1 for details as to the different CA approaches CA_Indiv_, CA_LNSum_, CA_Default_.

Figure 3 summarizes the accuracy and precision of predicted CA PODs for the median individual compared with those obtained from the mixture experiments. In general, most EC_10_ and LAI values are located within 10-fold lines in Figure 3A and Figure 3B, respectively. Among three different methods for CA prediction, EC_10_ of the CA lognormal sum approximation (CA_LNSum_) shows the best correlation with the measured value, with the greatest accuracy and precision. However, predictions for the other two CA methods, CA_Indiv_ and CA_Default_, were quite similar, although CA_Indiv_ showed greater uncertainty for Expo-L, Expo-H, and RfD-L mixtures. As with the results above for individuals evaluated separately, the AC_50_-H mixture also has the lowest EC_10_ and LAI values, showing a greater-than-additive effect of almost 10-fold, and two POD mixtures have AIs greater than 1, demonstrating a slightly less-than-additive effect. The AI values across all CA approaches and mixtures range from 0.035 to 3.71.

**Figure 4:**
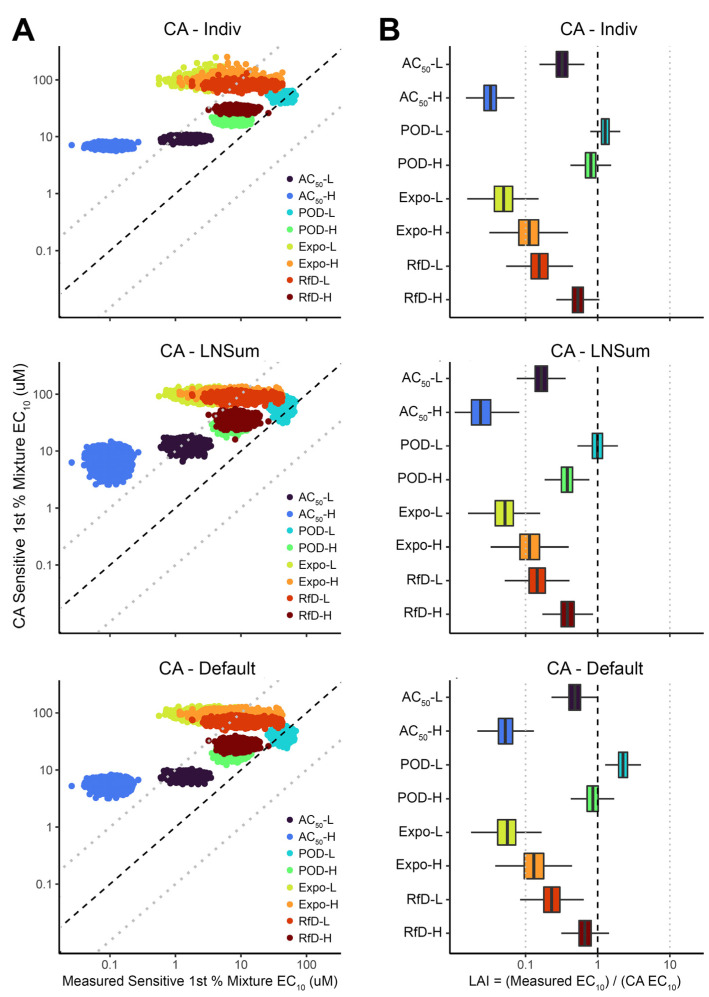
Comparison of mixture points of departure (POD = EC_10_) measured and predicted by different concentration addition (CA) approaches for sensitive (1st percentile) individual in the population. (**A**) Scatter plot of Bayesian posterior samples of measured and CA-predicted EC_10_ for the population’s 1st percentile individual. (**B**) Posterior distribution of Loewe Additivity Index (LAI) for each mixture for the population’s 1st percentile individual, where LAI < 1 indicates greater-than-additive effects (“synergy”) and LAI > 1 indicates less-than-additive effects (“antagonism”). In both panels, dashed line indicates equality, and light dotted lines indicate 10-fold differences in either direction. See Section 2 and Figure 1 for details as to different CA approaches CA_Indiv_, CA_LNSum_, CA_Default_.

For the most sensitive first percentile, CA-predicted EC_10_ values had a pattern similar to that of the median individual, but were further shifted to larger values relative to measured EC_10_s (Figure 4A). Consequently, the LAI values are also generally smaller and have greater variation across mixtures (Figure 4B), though the POD-L and POD-H LAI values were still generally higher than those of other mixtures. The LAI values across CA values and mixtures range from 0.005 to 5.05, with most mixtures showing greater-than-additive effects averaging nearly an order of magnitude.

### 3.3. Concentration Addition Predictions for the Toxicodynamic Variability Factor (TDVF_01_) 

**Figure 5:**
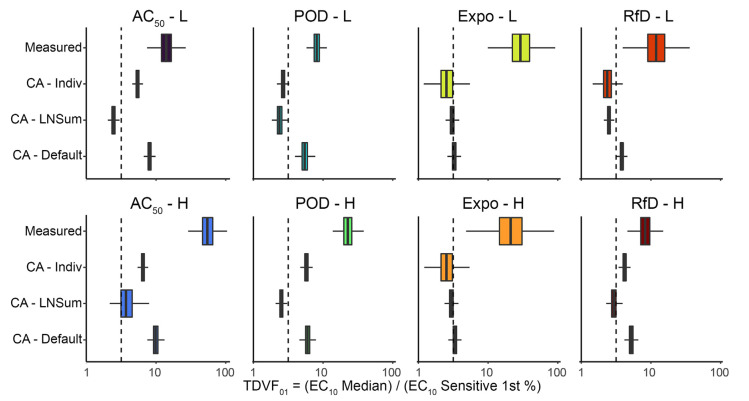
Comparison of toxicodynamic variability factor covering the sensitive (1st percentile) individual (TDVF_01_) measured and predicted by different concentration addition (CA) approaches. See Section 2 and Figure 1 for details as to different CA approaches CA_Indiv_, CA_LNSum_, CA_Default_. Vertical dashed line indicates the default uncertainty factor for toxicodynamic variability UF_H,TD_ = 10^½^.

Figure 5 compares the TDVF_01_, as the ratio of the median individual’s POD to the sensitive (first percentile) individual’s POD, between measured and CA-predicted values. Most measured TDVF_01_ values are about 10-fold or greater, and all CA approaches lead to under-prediction of population variability. Excluding Expo-L and Expo-H, where all CA approaches predicted similar TDVF_01_ ≈ 3, the general trend across other mixtures is CA_Default_ ≥ CA_Indiv_ ≥ CA_LNSum_. This trend across different mixtures is sensible because CA_Default_ essentially assumes the sensitive (first percentile) individual for one chemical is the same as the sensitive (first percentile) individual for any other chemical (i.e., sensitivity is perfectly correlated at the individual level), CA_LNSum_ assumes the sensitivity is randomly distributed across chemicals (i.e., sensitivity is uncorrelated at the individual level), and CA_Indiv_ incorporates the observed correlation (see Section 3.1, Figure 2), which is intermediate between the other two extremes. However, inter-chemical correlation of sensitivity is not sufficient in accounting for the under-prediction of the TDVF_01_; thus, whatever factors led to greater-than-additive effects at the individual level were also influencing the TDVF_01_.

### 3.4. Comparison of Loewe Additivity Index (LAI) across Cell Types 

**Figure 6:**
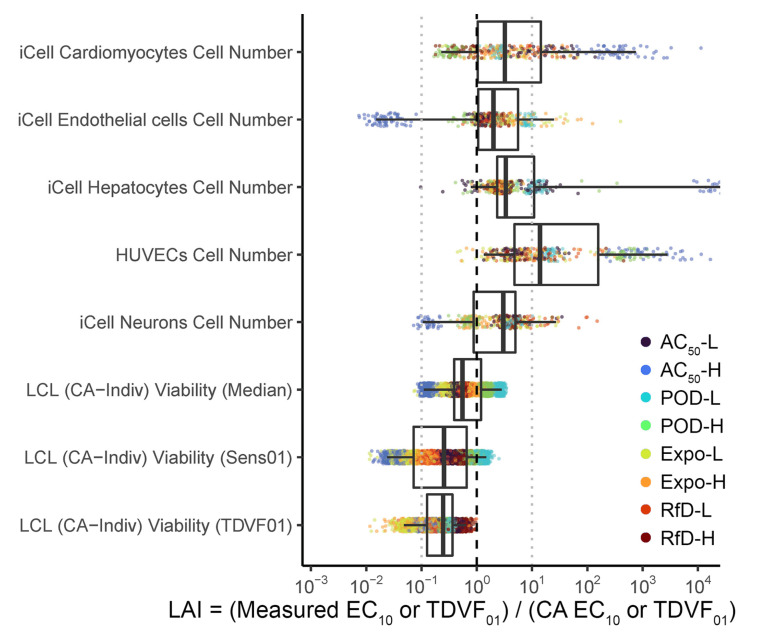
Comparison of Loewe Additivity Index (LAI) across cell types for cytotoxicity phenotypes. iCell and HUVEC results are from [30]; LCL results are those for CA_Indiv_ from the present analysis of data reported in [47]. LAI < 1 indicates greater-than-additive effects (“synergy”) and LAI > 1 indicates less-than-additive effects (“antagonism”). In both panels, dashed line indicates equality, and light dotted lines indicates 10-fold differences in either direction. Results for phenotypes from [30] and other CA methods from the present analysis are shown in Supplementary Figure S1.

We have found that for LCL data, CA approaches tend to underestimate POD and TDVF_01_ values, with LAI generally < 1 (Figure 3, Figure 4 and Figure 5). The same chemicals and defined mixtures were previously evaluated [30] using five different human cell types (iPSC-derived cardiomyocytes, endothelial cells, neurons, and hepatocytes, as well as human umbilical vein endothelial cells (HUVECs)), but each of these were from a single individual. Therefore, we compared the accuracy of CA, as quantified by the LAI, from these previous data with those analyzed here from LCLs. For cytotoxicity phenotypes (Figure 6), the LAI across all cell types is mainly between 0.1 and 10, indicating that CA predictions are within an order of magnitude of measured effects, though LCL values for LAI tend to be <1 while other cell types typically have LAI > 1 (Table 1). However, the 95% confidence interval on LAI reaches < 1 for all cell types except HUVECs.

When comparing across all cell types and diverse phenotypes (Supplementary Figure S1), these differences across cell types are, on average, similar in pattern to cytotoxicity, but iPSC-derived cells and HUVECs show much more variability in the accuracy of CA, with some phenotypes and mixtures having LAI << 0.1 and others having LAI >> 10. Additionally, the lower confidence bound across mixtures has LAI < 1 for all cell types and phenotypes except for two endpoints in HUVECs. Quantitatively, differences across mixtures and cell type/phenotype combinations accounted for 52% of the variance in log_10_LAI by two-way ANOVA, with variation across mixtures contributing 15%, variation across cell type/phenotype combinations contributing 37%, and a residual standard error of 10^0.6^.

## 4. Discussion

There have been numerous attempts to understand the complexity of risk assessment for mixtures and overcome concurrent limitations for mixture studies [63,64,65,66]. The variability of chemicals in mixtures with different compositions makes it challenging to estimate overall risks [67]. CA and IA are widely used to quantify the combined toxicity of a mixture using the toxicity data, when available, of individual substances in the mixture [68]. CA is based on an assumption that all components act on the same biological site (mechanism of action, mode of action, endpoint, or target tissue), and IA assumes that components in a mixture act independently from each other. Although neither of the two methods have been universally accepted as a unified approach for estimating the effects of chemical mixtures, for non-cancer endpoints, CA is more commonly applied due to the biological plausibility of additivity for common effects and is considered a default in the absence of adequate data for verifying alternative cumulative mixture models. Additionally, due to increasing interest in filling the gaps for chemical and mixture risk assessment with data, the importance of high-throughput and more biologically relevant NAMs has been highlighted, with efforts to replace animal experiments by implementing in vitro and in silico models for hazard and risk characterization [69]. Collecting experimental chemical-specific data on a large number of different chemicals and their mixtures using NAMs is a promising and effective way to fill data gaps and replace default assumptions.

The primary innovation of this study is the use of a human population-based in vitro model for both individual chemicals and mixtures to better understand population inter-individual variability in mixture effects, as there has been limited research on evaluating population variability in the additivity of responses to mixtures. We exposed 146 human lymphoblastoid cell lines from 4 subpopulations to 42 ATSDR priority chemicals and 8 defined mixtures to investigate population variability [47]. Lymphoblastoid cells are useful because they are relatively easy to culture and demonstrate comparable results to iPSC-derived models, which are known as reliable human cell types for in silico studies; further, they can be used to study population variability as an alternative to iPSC-derived models. Further, the lymphoblast cell line model is promising for the quantification of inter-individual differences for risk assessment, which has largely been limited to using a default uncertainty factor of 3 or 10, despite its importance in dose–response assessment to protect the sensitive population for risk-based decision making [45,46,70]. Of importance in our study is that we used 42 ATSDR priority list chemicals that do not necessarily share the same mode of action. Chemicals were selected from various classes for the experiments, and are thus directly testing the idea that additivity can be applied in the case of a common endpoint alone, similar to how the HI for dose addition in cumulative risk assessment is applied. Moreover, to account for population variability, we tested three different CA approaches for combining component-level population variability into a mixture-level population variability.

Our overall goal was to assess the influence of multiple factors, including genetics, cell type, phenotype, and mixture composition, on the accuracy of CA. With respect to genetics, we did find inter-individual variability in the performance of CA ranging up to two orders of magnitude. Thus, the use of isogenetic test systems may not be representative when combining components to estimate mixture effects, and argues for testing in a genetically diverse population-based paradigm. This is further supported by our finding that CA is more accurate when applied to the population median POD estimated with data from multiple individuals, and less accurate when applied to the “sensitive” individual in the population. Moreover, these results suggest caution when applying CA to assess population variability in a mixture, since the accuracy of CA is not uniform across the population and, thus, mixture TDVF values may be underestimated. We also found that mixture composition had a greater influence on CA accuracy than genetics within the same cell type and phenotype, again showing the need for caution in using CA for a particular mixture. On the other hand, the accuracy of CA was more variable across cell types and phenotypes than it was across mixtures. This is unsurprising, since cell viability and perturbation of functional readouts have very different mechanisms across cell types and phenotypes. 

Additionally, our study evaluated three different CA approaches in a population context. We found that for predicting the population median mixture POD, the three CA approaches gave similar results, though all were somewhat under-predictive. At the same time, predictions for the sensitive (first percentile) individual’s POD as well as the TDVF_01_ were more variable across CA approaches. These results can be explained by the three approaches making different assumptions as to the degree to which sensitive members of the population may respond to different chemicals. In particular, CA_Default_ assumes that sensitivity is the same across each component, CA_LNSum_ assumes that sensitivity is randomly distributed across components, and CA_Indiv_ uses the data on sensitivity of each individual to each component to build the distribution of sensitivities for the mixture. These assumptions have little impact on the central tendency of the population, but model the tails of the population variability distribution differently. Nonetheless, we also found that regardless of the approach, CA under-predicted the sensitive tail of the distribution by up to an order of magnitude. 

Our study has several important limitations. We used only in vitro data and did not include or compare them with in vivo data because these particular defined mixtures have not been tested in animal studies. Even though in vitro data may have limited utility for replicating all complex mechanisms and metabolism that may occur in a whole living organism, quantitative in vitro-to-in vivo extrapolation (IVIVE) is an emerging methodology to derive in vivo chemical-specific toxicity values from in vitro data [71]. We reason that while our results should be interpreted with caution with respect to the accuracy of CA, more generally, it is unlikely that large-scale in vivo datasets for CA of complex mixtures from diverse chemical classes will become available to address these issues directly. Still, it may be possible to conduct these evaluations in reverse, based on exposome-enabled epidemiology [72] where mixture effects are observed in human populations, and then attempts are made to reproduce those effects using NAMs. Additionally, only LCLs have been evaluated for CA in a population setting; the other models in which this analysis has been performed are based on a single individual for other cell types. Among reproducible cell types, only cardiomyocytes have been available in a population context [73], so a similar analysis using iPSC-derived cardiomyocytes may be a useful future point of comparison.

Finally, it may be that our findings of possible greater-than-additive effects, in many cases, might reflect in vitro bioavailability in a mixture context, such as saturation of binding in the presence of a mixture [70]. Moreover, if protein binding is an issue, differences in protein content across different media types, as compared to human plasma, will make it challenging to extrapolate these data to in vivo. While, ideally, cellular concentrations of each component would be measured in both single-component and mixture experiments, this is a technically challenging approach. Alternatively, computational mass balance models such as [74,75,76,77] have been proposed as a substitute but, unfortunately, computational bioavailability estimates were only available for 34 out of 42 of our components in a single-chemical setting (nominal depletion and cellular enrichment factors in Supplemental Table S1). However, based on the results for those 34 chemicals calculated using EAS-E Suite Ver.0.95 [78], only 7 chemicals showed non-linearity indicative of saturation of bioavailability in the range of component PODs (Supplemental Figure S2A), and for only two of these chemicals were the mixture PODs within the saturated bioavailability range (Supplemental Figure S2B). While imperfect, these results suggest that saturation is unlikely to be a reason for the departures from additivity. Nonetheless, to assess the sensitivity of our results to saturation in bioavailability, we recalculated the individual-level CA truncating all PODs at the concentration corresponding to saturation (Supplemental Figure S3), and found patterns nearly identical to our primary results in Figure 2. It is possible that more sophisticated bioavailability calculations or measurements, including those in a mixture setting, would improve the performance of CA, as we have previously shown that plasma protein binding changes in a mixture setting compared to individual-chemical settings [70].

In sum, this study demonstrates the presence of inter-individual variability in CA, with evaluations of how well CA can predict mixture effects from component effects. A number of significant conclusions can be drawn from our results. First, the accuracy of CA predictions has greater variation between different mixtures than across individuals in the population. Thus, the composition of mixtures influences the variation of POD estimates more significantly than inter-individual differences. Second, CA accuracy is highly variable and not always health-protective, but CA predictions are usually no more than 10-fold under-protective, both for the population central tendency as well as for the sensitive members of the population. The high variability in CA accuracy supports the need for toxicity and bioactivity evaluations on the whole mixture, rather than only on components. Our data, using a NAMs approach, show a sensible path to deriving such estimates using an empirical data-driven approach rather than relying on defaults. Moreover, these results suggest that in the absence of whole-mixture data, risk characterization benchmarks based on additivity, such as the HI, may need to be revised (i.e., reduced) by about 10-fold (i.e., HI = 0.1 rather than HI = 1.0 as the screening threshold) in order to ensure that greater-than-additive effects are protected. This idea has recently been proposed in the form of a Mixture Assessment Factor (MAF), which would have the same effect of reducing the risk characterization benchmark [79,80,81]. 

## 5. Conclusions

Overall, in a series of studies, we have demonstrated how human cell-based, in vitro NAMs can be highly informative in characterizing effects of complex mixtures with diverse chemical components. We have previously found that the usual assumption of CA exhibits a high degree of variability in its accuracy in predicting mixture bioactivity across multiple mixtures, multiple cell types, and multiple phenotypes. The present study not only further corroborates these conclusions, but also extends them by including variability across lymphoblastoid cells derived from a genetically diverse population of almost 150 individuals. We found that CA is more accurate in predicting the POD for the population median than for more sensitive individuals; hence, estimates of toxicodynamic variability of a mixture may be underestimated by CA of the population variability of components. Because the accuracy of CA varies widely from under-predicting mixture effects by more than 10-fold to overpredicting by more than 100-fold, the best strategy would appear to be direct testing of whole mixtures. However, in the absence of such data, our results support the need for cumulative risk assessment conducted using default additivity assumptions to implement more stringent benchmarks by up to 10-fold in order to ensure public health protection of mixture effects.

## Figures and Tables

**Table 1:** Summary statistics of Loewe Additivity Index (LAI, median posterior estimate, and 95% CI) across cell types for cytotoxicity phenotypes.

Cell Type ^1^	Phenotype ^2^	LAI [95% CI] ^3^
LCL (CA-Indiv)	Viability (Median)	10^−0.26 [−0.96, 0.45]^
LCL (CA-LNSum)	Viability (Median)	10^−0.23 [−0.76, 0.43]^
LCL (CA-Default)	Viability (Median)	10^−0.37 [−1.06, 0.25]^
LCL (CA-Indiv)	Viability (Sens01)	10^−0.59 [−1.62, 0.17]^
LCL (CA-LNSum)	Viability (Sens01)	10^−0.77 [−1.79, 0.09]^
LCL (CA-Default)	Viability (Sens01)	10^−0.44 [−1.48, 0.43]^
LCL (CA-Indiv)	Viability (TDVF_01_)	10^−0.61 [−1.32, −0.19]^
LCL (CA-LNSum)	Viability (TDVF_01_)	10^−0.8 [−1.34, −0.31]^
LCL (CA-Default)	Viability (TDVF_01_)	10^−0.52 [−1.18, −0.05]^
iCell Cardiomyocytes	Cell Number	10^0.51 [−0.64, 2.88]^
iCell Endothelial cells	Cell Number	10^0.3 [−1.82, 1.4]^
iCell Hepatocytes	Cell Number	10^0.52 [−0.11, 5.01]^
HUVECs	Cell Number	10^1.15 [0.13, 3.45]^
iCell Neurons	Cell Number	10^0.48 [−0.98, 1.43]^
iCell Cardiomyocytes	Other phenotypes	10^0.25 [−1.14, 1.62]^
iCell Endothelial cells	Other phenotypes	10^0.53 [−2.59, 1.85]^
iCell Hepatocytes	Other phenotypes	10^0.67 [−1.35, 3.34]^
HUVECs	Other phenotypes	10^0.58 [−1.95, 2.1]^
iCell Neurons	Other phenotypes	10^0.4 [−1.02, 1.43]^

^1^ See Section 2 and Figure 1 for details as to different CA approaches CA_Indiv_, CA_LNSum_, CA_Default_. ^2^ Median = population median individual, Sens01 = sensitive (1st percentile) individual, TDVF_01_ = toxicodynamic variability factor for 1st percentile individual. “Other phenotypes” are an aggregate of all other phenotypes reported in [30] other than “Cell Number.” Results for all separate phenotypes are contained in Supplementary Table S2. ^3^ LAI < 1 indicates greater-than-additive effects (“synergy”) and LAI > 1 indicates less-than-additive effects (“antagonism”).

## Data Availability

The data presented in this study are openly available in https://github.com/Suji-Jang/LCL-2022.

## Supplementary Materials

The following supporting information can be downloaded at: https://www.mdpi.com/article/10.3390/toxics10100549/s1, Figure S1: LAI comparisons for different CA approaches across various in vitro models, Figure S2: Summary of concentrations where computational modeling indicates saturation of bioavailability. For each component chemical (panels), horizontal grey line is 100 μM (maximum concentration in single-component experiments), horizontal dashed line is concentration where saturation begins. Circles represent the POD concentrations of component chemicals in each individual experiment (A) and each mixture (B) and different colors represent proportional [light green] versus saturated [dark purple] bioavailability, Figure S3: Comparison of mixture points of departure (POD = EC_10_) measured and predicted by concentration addition (CA) for each individual cell line (same as Figure 2), but truncating PODs at the saturating concentrations (shown in Figure S2), Table S1: Excel file with three tabs having information on chemical components: A—Chemical identification information, B—Mixture concentrations in μM, C—Mixture fractions, Table S2: LAI values for each phenotype from various models and other CA methods from present analysis.

## Abbreviations

CA, concentration addition; DA, dose addition; IA, independent action; HI, hazard index; NAMs, new approach methodologies; ATSDR, Agency for Toxic Substances and Disease Registry; AC_50_ high/low, active concentration 50%; DMSO, dimethyl sulfoxide; EC_10_, effective concentration at which a 10% change in cytotoxicity in comparison to vehicle; Expo high/low, exposure; HPV, high-production volume chemicals; iPSC, induced pluripotent stem cells; LCLs, lymphoblast cell lines; HUVEC, human umbilical vein endothelial cells; MCMC, Markov chain Monte Carlo; PAHs, polycyclic aromatic hydrocarbons; POD, point of departure; RfD high/low, reference dose; TDVF, toxicodynamic variability factor; GM, geometric mean; GSD, geometric standard deviation; LAI, Loewe Additivity Index; ANOVA, analysis of variance; MOE, margin of exposure.

## Appendix A

Although in vitro bioavailability is a critical aspect of in vitro-to-in vivo extrapolation, when evaluating CA, it turns out the LAI statistic for evaluating the accuracy of CA is independent of differences in bioavailability across components as long as, for each component, it is constant across concentrations and mixtures.

Specifically, consider *CEF_k_* to be the cellular enrichment factor, defined as the ratio between cellular and nominal concentrations, for chemical *k*. The individual component chemical nominal POD*_k_* can be converted to a cellular value.

POD*_cells,k_* = *CEF_k_* × POD*_k_* (A1)

Similarly, the nominal POD*_m_* of a mixture can be converted to a cellular value

POD*_cells,m_* = POD*_m_* × Σ *CEF_k_* × *f_k_*
= POD*_m_* × *CEF_m_*. (A2)

where we define the mixture cellular enrichment factor as

*CEF_m_* = Σ *CEF_k_* × *f_k_* (A3)

Furthermore, the fractional cellular concentration in each component is

*f_cells,k_* = *CEF_k_* × *f_k_* ÷ *CEF_m_* (A4)

Thus, CA-predicted POD is calculated by

1/POD*_cells,CA_* = Σ *f_cells,k_*/POD*_cells,k_* = Σ *f_k_*/(*CEF_m_* × POD*_k_*) (A5)

where we used Equations (A1) and (A4), cancelling the common factor *CEF_k_*. The LAI based on cells is

LAI*_cells_* = POD*_cells,m_*/POD*_cells,CA_* = [POD*_m_ CEF_m_*] × [Σ *f_k_*/(*CEF_m_* × POD*_k_*)] = [POD*_m_*] × [Σ *f_k_*/POD*_k_*] = POD_m_/POD_CA_ = LAI (A6)

which is the same as LAI without considering cellular enrichment.
