# Supplementary material for: Cumulative Risk Meets Inter-Individual Variability: Probabilistic Concentration Addition of Complex Mixture Exposures in a Population-Based Human In Vitro Model
Source: Toxics. 2022 Sep 20;10(10):549. doi: 10.3390/toxics10100549 (PMC9611413; doi:10.3390/toxics10100549)
Supplement: Supplementary file 1 [file toxics-10-00549-s001.zip › toxics-1850534-supplementary.pdf]

toxics

MDPI

Supplementary Materials: Cumulative Risk Meets Inter-Individual Variability: Probabilistic Concentration Addition of Complex Mixture Exposures in a Population-Based Human In Vitro Model

Suji Jang, Lucie C. Ford, Ivan Rusyn and Weihsueh A. Chiu

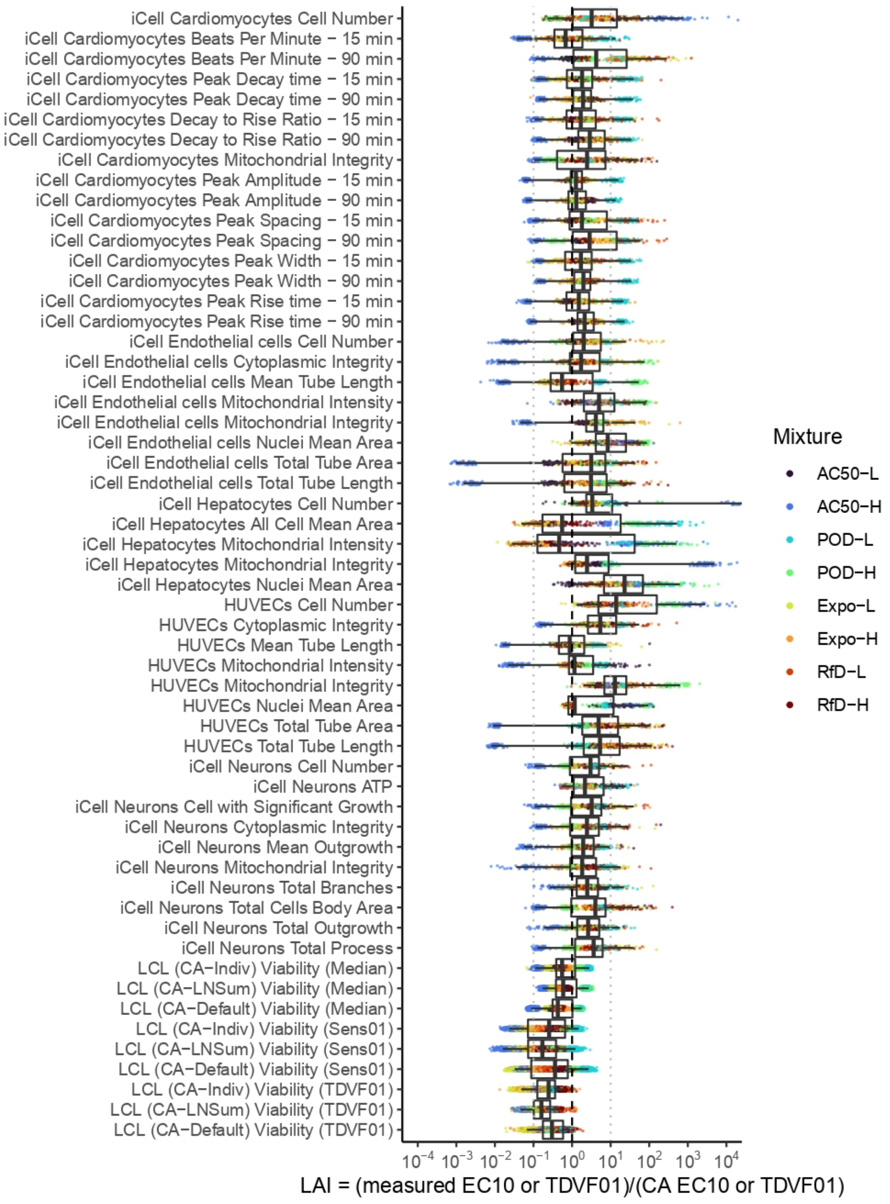

Figure S1. LAI comparisons for different CA approaches across various *in vitro* models

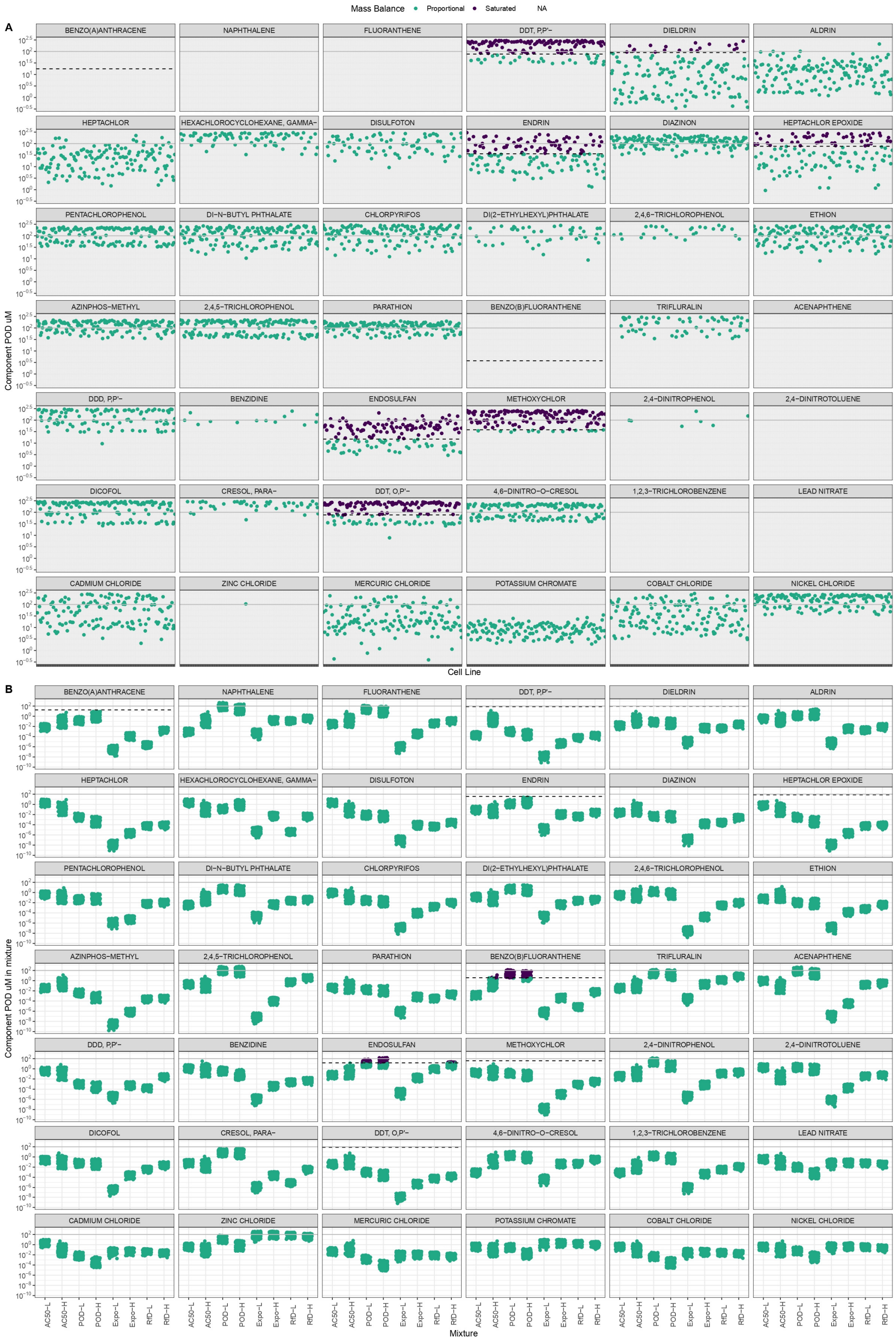

Figure S2. Summary of concentrations where computational modeling indicates saturation of bioavailability. For each component chemical (panels), horizontal grey line is 100 M (maximum concentration in single component experiments), horizontal dashed line is concentration where saturation

begins. Circles represent the POD concentrations of component chemicals in each individual experiment **(A)** and each mixture **(B)** and different colors represent proportional [light green]) versus saturated [dark purple] bioavailability.

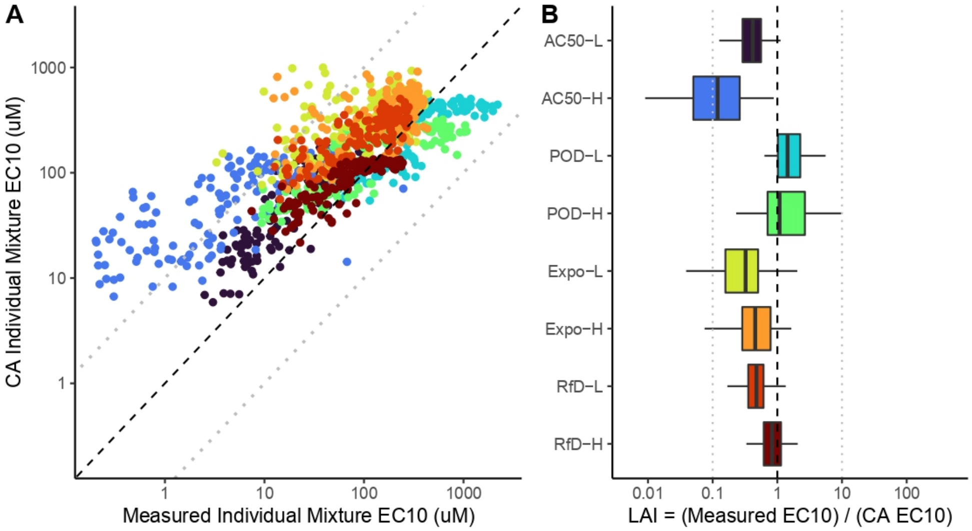

Figure S3. Comparison of mixture points of departure (POD = EC10) measured and predicted by concentration addition (CA) for each individual cell line (same as Figure 2), but truncating PODs at the saturating concentrations (shown in Figure S2)
